# HIV-Associated Interactions Between Oral Microbiota and Mucosal Immune Cells: Knowledge Gaps and Future Directions

**DOI:** 10.3389/fimmu.2021.676669

**Published:** 2021-09-20

**Authors:** Modupe O. Coker, Cristiana Cairo, Alfredo Garzino-Demo

**Affiliations:** ^1^Department of Oral Biology, School of Dental Medicine at Rutgers, Newark, NJ, United States; ^2^Department of Epidemiology, School of Public Health at Rutgers, Newark, NJ, United States; ^3^Institute of Human Virology, School of Medicine, University of Maryland, Baltimore, MD, United States; ^4^Department of Medicine, School of Medicine, University of Maryland, Baltimore, MD, United States; ^5^Department of Microbiology and Immunology, School of Medicine, University of Maryland, Baltimore, MD, United States; ^6^Department of Molecular Medicine, University of Padova, Padova, Italy

**Keywords:** immune, microbiome, oral, HIV, mucosal immunity

## Abstract

Even with sustained use of antiretroviral therapy (ART), HIV-infected individuals have an increased risk of systemic comorbid conditions and oral pathologies, including opportunistic infections, oral mucosal inflammation, and gingival and periodontal diseases. The immune-mediated mechanisms that drive this increased risk, in the context of sustained viral suppression, are unclear. HIV infection, even when controlled, alters microbial communities contributing to a chronic low-grade inflammatory state that underlies these non-HIV co-morbidities. The higher prevalence of dental caries, and mucosal and periodontal inflammation reported in HIV-infected individuals on ART is often associated with differentially abundant oral microbial communities, possibly leading to a heightened susceptibility to inflammation. This mini-review highlights current gaps in knowledge regarding the microbe-mediated oral mucosal immunity with HIV infection while discussing opportunities for future research investigations and implementation of novel approaches to elucidate these gaps. Interventions targeting both inflammation and microbial diversity are needed to mitigate oral inflammation-related comorbidities, particularly in HIV-infected individuals. More broadly, additional research is needed to bolster general models of microbiome-mediated chronic immune activation and aid the development of precise microbiota-targeted interventions to reverse or mitigate adverse outcomes.

## Introduction

The impact of HIV infection on the immune system results in a high incidence of opportunistic infections, cancers, and various end-organ manifestations (1), in ways that go well beyond the direct effects of infection on target cells. Rapid loss of CD4^+^ T cells in the gut mucosa results in loss of barrier integrity, with translocation of microbial products, including lipopolysaccharides (LPS) (2), to the systemic circulation. Such products induce high levels of inflammation, which fuels further HIV replication, and infers damage on end organs (2–6). While this vicious circle has been documented in great detail in the gut mucosa both in humans and in pathogenic models of SIV infections in macaques (2), much less is known about the oral mucosa, despite the occurrence of oral opportunistic infections, cancers, and other oral manifestations of HIV infection. While antiretroviral therapy (ART) partially restores CD4^+^ T-cell counts, and can suppress HIV viremia at undetectable levels, residual inflammation and disease manifestations continue to be observed in persons living with HIV (PLWH), including oral symptoms. The complex interplay between HIV and the immune system has also important effects on the microbiome, in the gut and orally, which itself may contribute to pathogenesis. Here, we review what is known about this interplay, what knowledge is lacking, and potential interventions and amelioration strategies.

Key interactions between microbiota and immune system have been reported in infectious diseases, autoimmune conditions, and cancer (7). Most of the existing research has, however, been focused on the gut, providing evidence of an altered gut microbiome in association with several diseases. In contrast, little is known about oral mucosal immunity and microbiota, particularly in PLWH. The prevalence of non-communicable diseases, including caries (8–17), mucosal inflammation (18), gingivitis (18–20), periodontal disease (21–23), and oral mucosal inflammation in general (24, 25), is higher in PLWH than in uninfected individuals, suggesting a heightened susceptibility to multifactorial chronic inflammation that would compromise the integrity of tooth-supporting tissues. The disruption of host–microbe homeostasis in oral epithelial tissues contributes to disease progression of gingival and periodontal diseases. This disruption is marked by a shift in the composition of the polymicrobial oral community to a dysbiotic and often pathogenic community, which fuels hyperactivation of the immune system and inflammatory conditions. The oral mucosa directly links oral bacteria to the bone through the teeth. The oral microbiome is complex involving several niches in the oral cavity including the saliva, tongue, supragingival and subgingival plaque, gingiva-crevicular fluid, buccal cavity, and soft (mucosal) and hard tissues. Bacteria colonizing these distinct niches are known to play a role in systemic inflammation and periodontitis, but the process by which this occurs is not known (26). The increased risk of periodontal disease in HIV-infected adults (22) and growing evidence of increased gingival inflammation in HIV-infected children (9, 19, 27–30) are likely driven by an altered or weakened immune response to oral commensals and pathogens.

## Impact of HIV Infection on the Intestinal Mucosa

HIV infection is characterized by disruption of the intestinal immune barrier and microbial translocation of microbial products leading to immune hyperactivation (31). When HIV is transmitted *via* the gut mucosa, CD4+ T cells are lost in a short time span (2, 32). Among them, the IL-17 producing subset that is known as Th17 is selectively infected and depleted (2–6). IL-17 exerts its activity on epithelial cells, which express its receptor, eliciting several effects: 1) expression antimicrobial peptides (AMPs), including ß-defensins, S100A8/9, and lipocalin; 2) expression of cytokines and chemokines (to IL-6, G- and GM-CSF, and IL-8), which induce inflammation and activate neutrophils; and 3) tissue repair (33–35). Thus, IL-17 production, often in concomitance with IL-22, is a key contributor to tissue homeostasis and response to infection (36). If produced excessively, it can drive inflammation (37), but defects in production (or blockade with antibodies in therapy) lead to loss of mucosal integrity (34, 38). Loss of mucosal integrity results in microbial translocation, with bacterial products inducing a strong inflammatory response. Parallel observations have been made in non-human primate models infected with pathogenic SIV, while non-pathogenic SIV does not cause loss of Th17 cells and associated events (2–4, 39–41).

Besides Th17 cells, other subsets of resident lymphocytes are required for the maintenance of mucosal homeostasis (42, 43). Among them, mucosal associated invariant T (MAIT) cells are significantly decreased or dysfunctional in PLWH, and ART only enables partial recovery of these subsets (44–51).

MAIT cells are a subset of innate-like T cells known to have broad and potent antimicrobial activity in response to microbial metabolites of vitamin B2 (52–54) and innate cytokines (IL-12 and IL-18) (55). Since these stimuli are reportedly elevated following microbial translocation (56), microbial translocation is hypothesized to directly contribute to the loss of circulating MAIT cells by causing hyperactivation and exhaustion (57, 58). Increased proliferation of MAIT cells (measured by Ki67) was reported in macaques after infection with SIV or SHIV (51, 59). While no MAIT depletion was observed in infected pigtail macaques (59), decreased MAIT frequencies in peripheral blood, mesenteric lymph nodes, and BAL of SIV-infected rhesus macaques appeared to be caused by increased cell turnover and were not the result of caspase 3-mediated apoptosis (51). Factors impairing the maintenance of IL-17 secretion are also thought to contribute to the depletion of MAIT cells and other IL-17-producing subsets in HIV chronic infection (51).

Therefore, even early in HIV infection, mucosal immunity is dramatically upended. Both physical and chemical barriers (such as AMPs) are decreased. This upheaval is reflected also in the composition of the microbiome, with dysbiosis, which itself becomes a factor that might contribute to driving high levels of inflammation. Inflammation, besides damaging organs and systems, also drives HIV replication, establishing a vicious circle of inflammation/damage/HIV replication (2–6, 39–41). While early adoption of ART preserves to some degree mucosal integrity, residual inflammation is observed even in PLWH undergoing therapy (60–62).

While the rapid disappearance of gut Th17 cells in PLWH is not clearly attributed to preferential HIV infection (2, 63, 64), it is very likely that microbial dysbiosis contributes significantly to Th17 and MAIT cell perturbation. In fact, several components of the intestinal microbiota influence cell-mediated immune response and gut dysbiosis is known to alter the homeostasis of intestinal MAIT and Th17 cells (43, 65–67). In HIV-infected macaques, gut dysbiosis resulted in an altered Th17 profile even in peripheral blood (68). Several studies suggest that PLWH have a dysbiotic gut microbiome with enrichment of Proteobacteria, *Prevotella*, Erysipelotrichaceae, and several pathobionts, and depletion of bacteria such as Lactobacillales, *Bacteriodes*, and short chain fatty acid (SCFA) producers, particularly in viremic subjects (69, 70). However, the composition of the microbiome of some ART-treated subjects was relatively similar to HIV uninfected controls in some other studies (71–76). The dysbiosis correlated with plasma levels of the inflammatory cytokine IL-6, and with activation of the kynurenine pathway, a known marker of disease progression (75). In particular, a study of a group of subjects with long-term ART controlled HIV infection showed gut microbiome dysbiosis with decreased levels of beneficial butyrate-producing taxa; the dysbiosis was associated with high levels of inflammation. Further, the gut microbiome of the PLWH was enriched in *Fusobacteria, Lactobacillus*, and Bifidobacteriales, which are typically associated with oral microbiome, possibly suggesting a loss of compartmentalization. Levels of *Prevotella*, although not differentially present in PLWH, negatively correlated with CD4+ T-cell counts (77). Dysbiosis could also be an outcome of Th17 cell depletion in the context of HIV infection. AMPs, which are produced when IL-17 binds to receptors on epithelial cells, are a key component of innate immunity on mucosa. They contribute to mucosal integrity, having co-evolved with mucosal microbiome, protecting the host against pathogenic infections (78, 79). Therefore, decreased production of IL-17 due to HIV infection is predicted to result in impaired production of AMP, loss of mucosal integrity, and dysbiosis.

## Oral Microbiome in HIV

While growing evidence suggests that in the ART era, PLWH continue to experience oral inflammation-associated and/or immunodeficiency-related infections (80), few studies in comparison to the gut studies have comprehensively characterized the oral microbiota in the context of HIV exposure, infection, and treatment (26, 81–91). PLWH have increased levels of oral mucosal inflammatory markers (92, 93), as compared to HIV-uninfected subjects, suggesting likely changes in the oral bacterial composition. Similar to the gut, evidence suggests HIV infection impacts the composition of the oral microbiome with differentially abundant taxa when compared to uninfected populations; however, findings to date are varied and inconsistent. Some studies found no significant taxonomic differences (94–96), while others have reported differentially abundant taxa (81, 83–85, 87, 89, 91, 97). For the lingual microbiome, potentially pathogenic *Veillonella*, *Prevotella*, *Megasphaera*, and *Campylobacter* were enriched, while *Streptococcus* sp. were depleted (82). *Streptococcus mutans, Lactobacillus*, *Candida, Haemophilus parahaemolyticus, Actinomyces, Neisseria subflava* (91), and *Corynebacterium diphtheriae* (91) species were reported to be more abundant in saliva of PLWH individuals (82, 95, 98, 99). Several studies have observed a lower proportion of *Streptococcus mitis* in saliva of PLWH compared to the uninfected (91, 95, 98). A study on HIV-infected women found that in the infected group, the microbiome had higher representation of *Prevotella melaninogenica* and *Rothia mucilaginosa* (88). When compared to the perinatally exposed but uninfected, subgingival plaque of HIV-infected youth differed in abundance of disease-associated taxa (85). While some studies showed differences in microbiome compositions based on CD4 counts, the impact on other immune status markers was not evaluated (86, 88, 89).

Although ART has been implicated as the driver of these observed differences in the oral microbiome (87, 100), it is increasingly hard to isolate the direct impact of HIV with widespread availability of highly active ART. While data from a number of studies suggest a bidirectional relationship between pre-exposure prophylaxis (PrEP) and ART-specific regimens, and the vaginal and intestinal microbiota (101–103), little information is available with respect to the oral microbiome. A recent study (103) suggests that ART, especially non-reverse transcriptase inhibitors (NRTIs), have considerably more impact on microbiota composition and diversity in the gut, leading to dysbiosis, than in the oral cavity.

Patients with oral co-infections displayed lower abundance of *Veilonella parvula* (81, 82), while ART was associated with higher levels of *Neisseria* and *Haemophilus*. Recent 16S analyses a strong relationship between salivary microbiota and CD4 T cells in HIV-infected children, specifically a distinct oral microbial community with HIV infection and low CD4 counts (91). The abundance of *Streptococcus* and *Lactobacillus* correlated positively with CD4 counts, and negatively with viremia, suggesting an underlying protective effect of these taxa (88). However, a study of alpha microbial diversity that compared salivary and fecal microbiome in PLWH reported microbiome changes associated with ART only in the fecal microbiome (103). These inconsistencies highlight the need to standardize sample collection protocols, sample type (i.e., mucosal swab, saliva, supra- or subgingival plaque) or other experimental variables that may have biased the results (103). Aging is another important factor that was associated with increased intra-sample microbiome diversity regardless of HIV status (94). Several host factors including genetics and immune status play important roles in the colonization of pathogenic bacteria and consequently contribute to disease outcomes (86), and need to be considered when addressing this question. Further, the impact of several other confounding factors (including age, sex, dentition, oral hygiene, periodontal disease, sex, salivary flow, body mass index, diet, cigarette smoking, antibiotic use, and the type and site of specimen collection) is an important point of consideration for future studies.

While phylogenetic approaches such as 16S sequencing are able to identify taxa that are unculturable within an ecological framework, species/strain resolution is often poor and their functional roles can only be inferred. Similarly, 16S studies do not assess the composition of the mycobiome, that may interact with, and influence, the microbiome (104). Results of a study that assessed mycobiome and microbiome in smokers and non-smokers. The study reported lower alpha diversity of the mycobiome in HIV-infected smokers than HIV-infected non-smokers, while richness of the microbiome in HIV-infected smokers was less than that of uninfected smokers, suggesting complex interactions between mycobiome and microbiome in different health conditions (83). Whole genome metagenomics allow for detailed investigations into oral microbial community diversity (both intra- and inter-sample), composition and function, yet there are few available studies regarding the relationship between the function of the oral microbiota and HIV. Therefore, additional studies are needed to clarify this complexity. The synergistic impact of fungal involvement, including *Candidiasis*, on the mucosa plays an important role in mucosal immunity. This is particularly important as candidiasis was one of the most common HIV-associated oral lesions prior to HAART initiation and was often pathognomonic for disease progression. Colonization of *C. albicans* and *C. dubliniensis* are the most prominent taxa observed in high abundance in saliva of HIV infected individuals. While incidence of oral candidiasis typically declines after HAART initiation, recent evidence suggests that the impact of HIV/HAART on the mycobiome is modest but not more considerable than other factors such as sex (105).

There is a dearth of studies focused on the gene expression and metabolic function of the oral bacterial communities with HIV infection. This is critical as recent evidence suggests significant functional redundancy such that even if communities differ in abundance, there is an inherent stability in ecologic function. Bacteria of the oral microbiome release metabolites—lipids, nucleic acids, polyuronic acids, proteins, and extracellular polymeric substances and microbial production that serve several functions. Worthy of note is the production of SCFA and tryptophan. SCFA are immunomodulatory products that have several effects on the oral epithelial barrier, and could represent the link between the bacterial communities and the immune system. Butyrate is a SCFA notorious in the oral environment for its deleterious impact on the gingiva and periodontium. Some potential mechanisms by which butyrate elicits their effects include cell apoptosis and upregulation of proinflammatory cytokines and modulation of the proteins in intercellular junctions (106, 107). The activation of the tryptophan metabolism pathway by the enzyme indoleamine 2,3 dioxygenase (IDO), which is expressed in macrophages and dendritic cells, produces kynurenine and other metabolites, which have immunomodulatory effects. The kynurenine/tryptophan ratio (KTR) is considered a surrogate marker of IDO activity, and is associated with immune activation (75, 108–114). Increased KTR have been consistently reported in PLWH (75, 108–114).

## Impact of HIV on Oral Mucosal Immunity

A predominant portion (~80%) of the oral cavity consists of oral mucosal surfaces, therefore presenting an extensive area for microbial attachment (115). Several research studies have reported mucosal immune cell dysfunction and its interaction with the oral microbiome, in the context of various chronic inflammatory diseases (116, 117). However, to our knowledge, none has comprehensively evaluated the contribution of the oral microbiota to mucosal immune perturbation in PLWH. Our understanding of the impact of HIV on the distribution and function of immune cells in the oral mucosa, the mechanism(s) for chronic oral inflammation and its role in increasing the risk for oral diseases, is limited. While there is growing evidence of a higher prevalence of oral disease in PLWH (24, 25), it is unclear how HIV, even in the context of suppressed viral replication, heightens susceptibility to oral mucosal inflammation.

Secretory immunoglobulin A (SIgA) antibodies in saliva are considered the first line of defense against pathogens present in the oral cavity. SIgA and other salivary antimicrobial systems also act against periodontopathic and cariogenic consortia by limiting adherence of pathogens and pathobionts to the mucosa (118, 119). These oral pathogens include the main cariogenic agent—*Streptococcus mutans*. SIgA plays an important role in the homeostasis of the oral microbiota as focus of much research in the last two decades has been on the development of a caries vaccine to stimulate induction of IgA responses in saliva (120, 121). In HIV+ individuals, dysregulation of CD4 T-follicular helper cell function greatly limits/impairs Ig class switching in subepithelial B cells, which results in a significant reduction of IgG and SIgA in mucosal fluids (122, 123). This decline may contribute to a perturbed composition of mucosal microbiome and to the compromise of mucosal barrier integrity.

**Figure 1:**
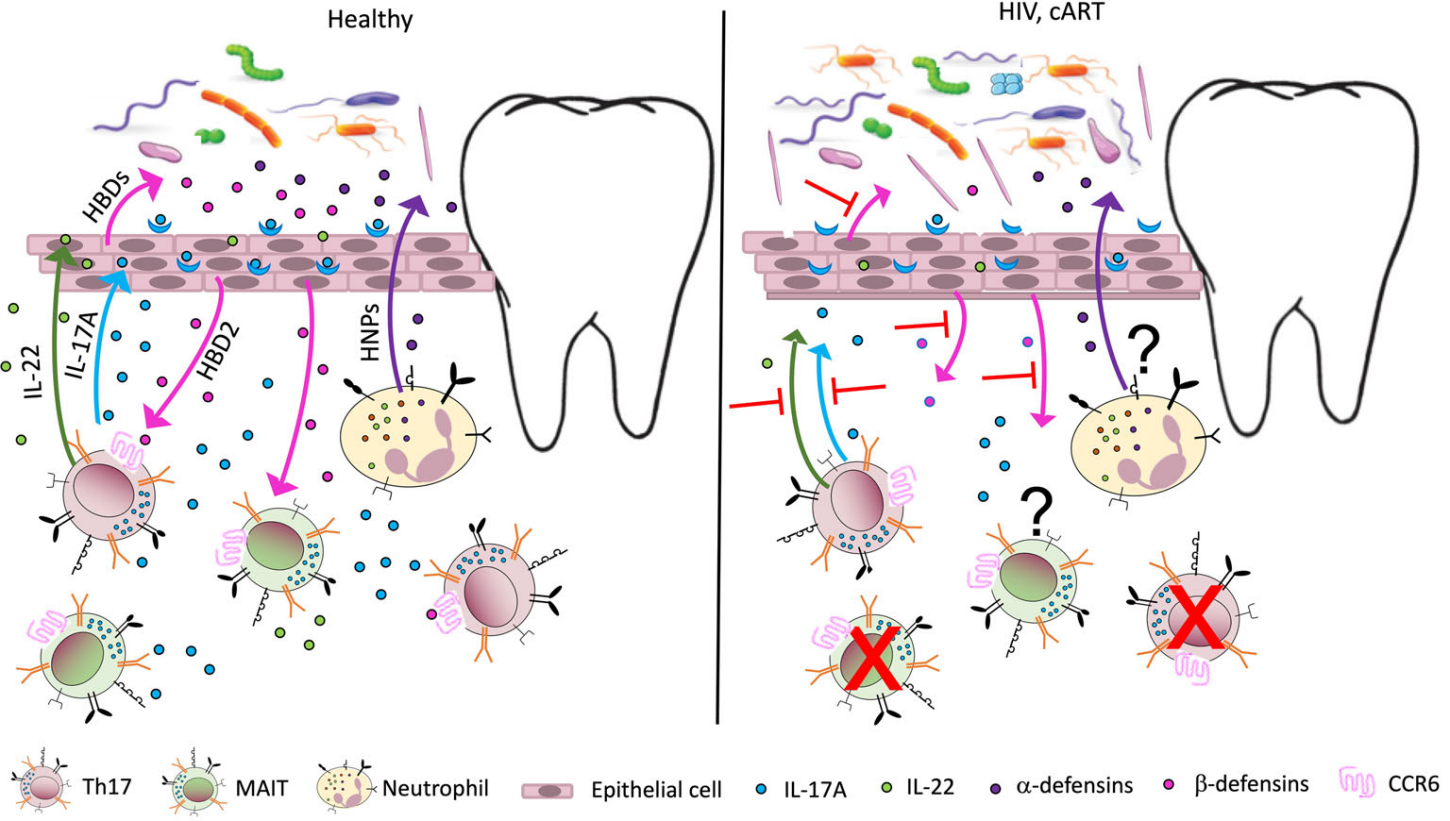
Potential immune perturbation affecting the oral mucosa in HIV-infected individuals/ PLWH. In healthy, uninfected individuals (left panel), Th17 cells, MAIT cells, and neutrophils contribute to mucosal homeostasis by producing various soluble factors involved in mucosal integrity. Th17 and MAIT cells secrete IL-17, a cytokine with antifungal and antibacterial function, which also acts on epithelial cells to induce secretion of antimicrobial peptides called human β defensins 1–3 (HBDs). Among them, HBD2 binds the receptor CCR6, expressed by Th17 and MAIT cells, with potential cytoprotective effects. Th17 cells also produce IL-22, important for tissue repair, while neutrophils are responsible for the secretion of another class of antimicrobial peptides, the human α defensins or human neutrophil peptides (HNPs). The antimicrobial peptides contribute to the homeostasis of the mucosal microbiome, promoting oral colonization of advantageous bacterial species. In PLWH (right panel), a large number of Th17 cells in the gut mucosa are lost due to active infection. The number of MAIT cells also declines in peripheral blood, possibly due to activation induced cell death. A decline in Th17 and MAIT cells may occur to some extent in the oral mucosa, leading to decreased levels of IL-17, IL-22, and HBDs. This imbalance may contribute to increased inflammation and perturbed microbiome in the oral mucosa (dysbiosis), increasing the risk of oral disease. The effects of antiretroviral therapy (cART) on oral inflammation and dysbiosis are unclear.

As mentioned above, HIV infection disproportionately affects Th17 cells (2, 6, 124) (**Figure 1**). Human β defensin 2 (hBD2) is not detectable in the oral mucosa of PLWH but is robustly expressed in HIV-uninfected controls (125). This defensin is of particular interest because it binds to CCR6, a shared chemokine receptor expressed on Th17 and MAIT cells (126). We also reported that hBD2 selectively protects CCR6+ CD4 T cells from infection (127, 128). Therefore, AMP could be at the center of the mechanism underlying the effects of HIV infection on Th17 cells resulting in loss of mucosal integrity and dysbiosis. Oral epithelial barrier function and mucosal immunity clearly depends on interactions between commensal microbiota and pathogens with toll-like receptors on epithelial cells (129).

As observed in the gut, the expression of AMPs is lower in oral mucosa of HIV-infected individuals undergoing ART, as compared to HIV-seronegative controls (125). AMPs have been shown to promote targeted killing of specific pathogenic taxa (130), so with decreased levels, the immune system is further compromised. This state of impaired innate immunity could increase the risk of oral mucosal pathologies such as gingival and periodontal inflammation. Microbial changes observed after ART administration include decrease in salivary *Aggregatibacter*, *Prevotella*, and *Haemophilus* which could in turn drive pathogenesis or facilitate colonization of taxa that have been implicated in periodontal disease (*Porphyromonas, Prevotella melaninogenica, Rothia mucilaginosa* and *Fusobacterium* in saliva, and *Rothia dentocariosa, Fusobacterium, Streptococcus*, and *Prevotella* in plaque) (87, 103). As a keystone pathogen, *P. gingivalis* impairs host immune responses and represents a necessary but not sufficient microbe for development of periodontitis. These taxa should be considered in the pursuit of developing treatments to minimize HIV-associated periodontal disease.

Studies focused on identifying early immunology and microbiota differences that could lead PLWH to an increased susceptibility to chronic inflammatory conditions are needed. This data is highly relevant to human health, addressing the role of the oral microbiota on immune cell response. By targeting AMPs and specific immune cells, which are known drivers of immunomodulation with established relevance and therapeutic potential, there would be an improved understanding of how the oral microbiota influences immune pathology, informing novel interventions for a wide range of oral diseases including mucosal infections and cancer.

## Future Investigations and Therapeutic Approaches

Our understanding of the interaction between oral microbiota and oral mucosal immune cells is still evolving. Significant gaps remain with respect to mechanisms of influence by microbiota on immune homoeostasis, and *vice versa*. Given the bidirectional relationship between oral microbiota and mucosal immunity (97), understanding the functions of microbes involved in influencing immune compartments, their physio-pathologic consequences, and contribution to oral disease pathology is essential to inform preventive and therapeutic approaches. This complex relationship demonstrates a clear need for continued investigation in both animal and human studies. Investigating the interplay between immune cell subsets and bacterial communities would inform strategies to improve and facilitate mucosal homeostasis.

Studies aimed at identifying early immunological and microbial features with the potential to increase the risk of chronic inflammatory conditions in PLWH are needed. In particular, understanding the interplay between oral microbiota and mucosal immunity may identify targets with therapeutic potential, thus informing novel interventions for a wide range of oral diseases, including mucosal infections and cancer. While there is evidence of SIgA in the oral mucosa with HIV, its unique molecular properties and interactions with pathogens and microbial metabolites should be incorporated in further investigations of the oral mucosal immunity (131). The specific impact of HIV on oral mucosal immunity has significant implications for specific sub-groups and risk populations such at sero-discordant couples, men who have sex with men (MSM), the aging, and growing children and adolescents.

Given the gaps in knowledge, characterizing features and functions of the oral microbiota associated with HIV infection would bring us closer to understanding the interplay between the oral microbiota and oral mucosal immunity. Multi-dimensional and multi-parametric approaches are needed to investigate microbe-mediated interactions as well as identify the microbial properties and immune parameters key for oral mucosal homeostasis. Such approaches will provide insight into how the oral microbiota could be used to mitigate immune perturbation in HIV.

Probiotic approaches to prevent oral diseases have been in previously spotlighted. An example in caries disease treatment is the displacement of native *S. mutans* strains with *S. mutans* strains engineered to have low pathogenicity (132, 133). While the results support the efficacy of these strains as anti-caries probiotics, further studies in humans are required. Future high-quality randomized controlled clinical trials that demonstrate the efficacy of probiotics (134, 135), antimicrobial agents and procedures on oral immune functions, will expand the current paradigm focused on intestinal bacteria by comprehensively studying microbe-mediated immune cell responses relative to oral bacteria. Understanding how the interaction between immune system and microbiota contributes to co-morbidities would provide additional targets for intervention and drive the success of future clinical trials. Research focused on bolstering general models of microbiome-mediated chronic immune activation and aiding the development of precise microbiota-targeted interventions to reverse chronic inflammation are needed. Cytological experiments and metagenome and transcriptome analyses will further characterize the biological processes and the molecular changes of specific oral bacteria. Results from future research studies are likely to inform preventive and therapeutic interventions. Interventions targeting both inflammation and microbial diversity are needed to reduce the risk of oral inflammation-related comorbidities, particularly in PLWH (84), high-risk populations such as MSM, and even more critical in developmental phases in children where appropriate immune training and maturation has far-reaching complications (136).

## Conclusion and Perspectives

In this review, we have discussed the current status of research on gut microbiome and HIV and reviewed recent advances in our understanding of the interaction between the oral microbiota and mucosal immune system in PLWH. Many studies of the oral microbiota suggest that individual or singular pathogens are not observed as differentially abundant in classic oral diseases such as caries or periodontitis. Perturbations among relatively less-abundant microbes appear to drive dysbiosis. Review of studies highlight an altered pathological status of the microbial communities and the immune systems even with ART. However, much work is required for a clearer understanding of the mechanisms of interaction between oral bacteria and specific T-cell subsets and their function. Therefore, in the future, it is important to focus our attention on the how to approach therapeutically dysbiosis, and/or its metabolic/inflammatory consequences, to ameliorate oral symptoms and standard of living of PLWH.

## Author Contributions

MC conceived the mini-review. CC developed the figure and AG-D provided guidance in direction. All authors edited and approved submission of the final version paper.

## Funding

This work was supported in part by grant from the National Institutes of Health (NIDCR R01DE028154; NICHD U01HD092308).

## Conflict of Interest

The authors declare that the research was conducted in the absence of any commercial or financial relationships that could be construed as a potential conflict of interest.

## Publisher’s Note

All claims expressed in this article are solely those of the authors and do not necessarily represent those of their affiliated organizations, or those of the publisher, the editors and the reviewers. Any product that may be evaluated in this article, or claim that may be made by its manufacturer, is not guaranteed or endorsed by the publisher.
